# Post-COVID-19 mucormycosis complication: a black fungal infection

**DOI:** 10.11604/pamj.2022.43.205.30128

**Published:** 2022-12-23

**Authors:** Shivani Rajesh Uttamchandani, Pratik Arun Phansopkar

**Affiliations:** 1 Department of Musculoskeletal Physiotherapy, Ravi Nair Physiotherapy College, Datta Meghe Institute of Medical Sciences, Sawangi Meghe, Wardha 442001, Maharashtra, India

**Keywords:** COVID-19, mucormycosis, rehabilitation, physiotherapy

## Image in medicine

We are presenting the case of a 27 years old male patient who was tested COVID-19 positive on 24^th^ of April, 2021 with HRCT score 15/25 on the same day for which he was treated in a hospital in Yavatmal; followed by which he complaint of left side nasal obstruction since 1 week which was insidious in onset and was gradually progressing. Patient tested negative on 10^th^ May, 2021 and he was brought to Hospital in Sawangi Wardha, with complaints of left side swelling and bleeding from nose, hemifacial pain and periorbital swelling since 6 days. Redness of face, watering and discharge from left eye, diminished vision were noticed from past 4 days. Mouth opening was noticed to be 2 fingers, palate was hard, blackish swelling was present of 0.05×0.05 cm at medial left side. Impression on CECT PNS reveals pansinusitis (mucormycosis) and features of left orbital cellulitis. Drain was seen into the maxillary sinus (A,B). Patient was newly diagnosed with Type 2 Diabetes Mellitus Post COVID with Diabetic Keto Acidosis ketoacidosis. Debridement was done for the upper palate (C). Physiotherapy was initiated. Patient was given manual chest percussions and vibrations, pursed lip breathing and thoracic expansion exercise which was given to mobilize secretions, relieve shortness of breath, and reduce work of breathing and to increase air entry to the affected areas of the lung. Secretions were noticed to be brownish/blackish coloured. Also facial exercises were taught to reduce soft tissue bulk and fat attenuation.

**Figure 1:**
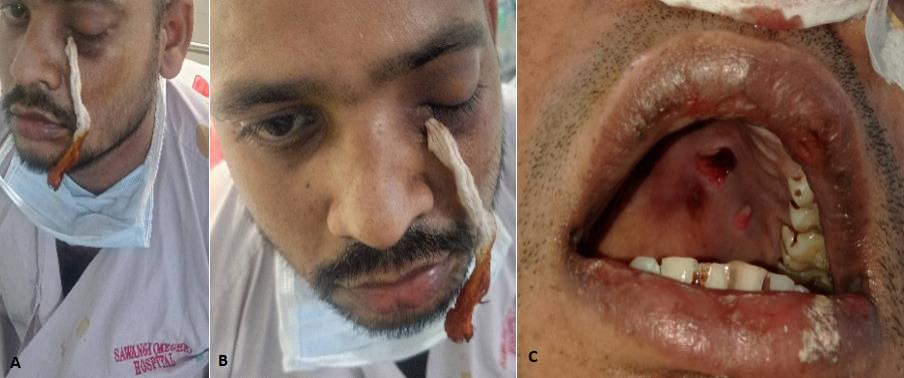
A,B) drain was seen into the maxillary sinus; C) debridement was done for the upper palate

